# Evaluation of Meat Quality of Local Pigeon Varieties in China

**DOI:** 10.3390/ani13081291

**Published:** 2023-04-10

**Authors:** Lingling Chang, Qingping Tang, Rui Zhang, Shengyong Fu, Chunyu Mu, Xinyue Shen, Zhu Bu

**Affiliations:** Poultry Institute, Chinese Academy of Agricultural Sciences, Yangzhou 225100, China

**Keywords:** pigeon, local varieties, meat quality, inosinic acid, amino acids, fatty acids

## Abstract

**Simple Summary:**

To evaluate the nutritional value of Chinese local pigeon varieties, this study examined the basic meat quality parameters and contents of conventional nutritional compositions, inosine, amino acids, and fatty acids of four Chinese local pigeon varieties (Taihu pigeon, Shiqi pigeon, Tarim pigeon, and Boot pigeon) and then compared them with those of White King pigeons. The results demonstrated that the meat of local breed pigeons had dark flesh, good water retention, high protein and inosine contents, a high proportion of essential amino acids and a low saturated fatty acid ratio compared with White King pigeons.

**Abstract:**

To evaluate the germplasm characteristics and nutritional value of Chinese native pigeon varieties, this study analyzed the nutrient composition of the meat of four Chinese native pigeon varieties and then compared them with those of the White King pigeon, which is the most commonly used in China. A total of 150 pigeons aged 28 d (squabs) of 5 breeds including Taihu pigeon, Shiqi pigeon, Ta-rim pigeon, Boot pigeon, and White King pigeon were selected for slaughter. The basic meat quality parameters and contents of conventional nutritional compositions, inosine acid, amino acids, and fatty acids were measured. The results showed that there were significant differences in flesh color (L*, b*), pH, and water loss rate of different breeds of suckling pigeons (*p* < 0.05). Compared with White King pigeons, four local breeds had dark breast meat and a low water loss rate. The protein contents of Taihu, Tarim, and Shiqi suckling pigeons were significantly higher than those of White King pigeons (*p* < 0.05). Taihu pigeons had the highest protein content, reaching 22.72%. The inosinic acid content of Tarim pigeons was the highest (1.31 mg/g) and was significantly higher than that of Shiqi pigeons, Boot pigeons, and White King pigeons (*p* < 0.05). There was no significant difference in the content of amino acids, the ratio of essential amino acids, and the ratio of umami amino acids in the meat of different breeds of pigeons (*p* > 0.05). The percentage of saturated fatty acids (SFAs) in the breast muscle of local breeding pigeons was significantly lower than that of White King pigeons (*p* < 0.05), and the percentages of lauric acid, palmitic acid, eicosanoic acid, and behenic acid in SFAs reached significant levels (*p* < 0.05). The content of eicosapentaenoic acid (EPA) in the meat of Taihu pigeons was significantly higher than that in other breeds. In conclusion, compared with the White King pigeon, the meat of local breed pigeons (Taihu pigeon, Shiqi pigeon, Tarim pigeon, and Boot pigeon) had dark flesh, good water retention, high protein and inosine contents, a high proportion of essential amino acids, and a low saturated fatty acid ratio. In addition, Taihu pigeons had the highest protein content (22.72%), monounsaturated fatty acids (44.58%), and EPA (0.47%) compared to other breeds.

## 1. Introduction

Pigeons are probably the first bird species to have been reared by humans at least 3000–5000 years ago [1]. In the past, pigeons were kept for emotional, religious, and cultural reasons, but were mainly used for sending messages [2]. When fresh meat was in short supply, pigeons were also kept by many households as a source of meat [3]. Egypt was the first country in the world to domesticate and breed pigeons and treat their meat as a delicacy [4]. With the continuous development of breeding technology, the United States announced the creation of the world’s first improved meat pigeon, the American King Pigeon, in the 1980s, which was an important milestone of the world meat pigeon industry and promoted the rapid development of pigeon breeding [5]. Then, the famous varieties of Homer pigeon, Carneau Pigeon, Luan pigeon, Mimas pigeon, Didan pigeon, and others were developed [6,7], and the pigeon breeding industry progressed successively in many countries such as France, Spain, China, Britain, India, and Bangladesh [8,9]. Although pigeon production may never rise enough to compete with commercial poultry as a major source of food, as a significant addition to the diet as well as a source of substantial income, pigeons and similar species of birds can play a pivotal role in third-world villages [10].

Pigeons have been reared in China for a long time, but only a few breeds are reared in the yard. In the 1980s, some breeds of pigeons with excellent performance were introduced from abroad to China, and the large-scale pigeon breeding industry began to develop [11]. After 40 years of development, China is the largest producer of pigeon meat in the world with an annual production of approximately 680 million squabs, accounting for approximately 80% of global production [12]. At present, there are approximately 30 varieties of meat pigeons in China, but most pigeon production relies on imported varieties [13]. According to the national catalog of livestock and poultry genetic resources of China (2021), there are only three certificated native pigeon varieties in China: Taihu pigeons, Shiqi pigeons, and Tarim pigeons [14]. The three varieties are distributed the lower-middle reaches of the Yangtze river, the Pearl River Delta, and the Xinjiang region respectively, and have contributed the largest amount of native pigeon breeding in China. Meanwhile, there are also some native varieties in preparation for resource identification (such as Boot pigeons), and the amount is only lower than the certificated varieties.

With the improvement in people’s living standards and the increasing demand for high-quality food materials, pigeon products no longer solely satisfy a demand for meat. Pigeon meat is characterized by a high nutritive value. Due to its low cholesterol and fairly high protein content, pigeon meat can be used as a valuable inclusive component of the human diet [15]. Native pigeon breeds are characterized by lower fattening, slaughter, and breeding yield parameters, but their advantages include good quality raw meat, lower feeding demands, natural resistance to bad environmental conditions, and higher resistance against illnesses and stress in comparison to regular industrial breeds [16]. Dariusz et al. [2] determined the carcass characteristics, physicochemical properties, and texture and microstructure of the meat and internal organs of carrier and King pigeons. There are few academic publications focused on the nutritional value of the meat of Chinese local pigeon varieties.

The purpose of this study was to evaluate the nutritional value of four local Chinese pigeon varieties, including the basic meat quality parameters and contents of conventional nutritional components, inosine, amino acids, and fatty acids, as compared to the White King pigeon, which is the largest feeder in the world. The results obtained allowed for determining and comparing the suitability for consumers of the meat from the examined pigeon varieties.

## 2. Materials and Methods

This study was conducted from January to June 2022 at the Poultry Institute, Chinese Academy of Agricultural Sciences (PI-CAAS), Yangzhou, China. The experimental procedures were approved by the Animal Ethics Committee of the PI-CAAS, and humane animal care and handling procedures were followed throughout the experiment (protocol number: PI-CAAS-2022-06).

### 2.1. Sampling

A total of 150 pigeons aged 28 d (squabs) of 5 breeds including the Taihu pigeon, Shiqi pigeon, Tarim pigeon, Boot pigeon, and White King pigeon were selected for slaughter. The birds were obtained from a feeding trial (mixture containing 14% protein and 12.3 ME/kg) that was uniform for all 5 breeds. The diet was cut off the night before the slaughter (fasting for 12 h), and the squabs were slaughtered at 8:00 the next morning to strip the breast muscles (including the pectoralis major and pectoralis minor). The right half of the breast muscles was used to determine the flesh color, pH, water loss rate, and shear force within 24 h. The left half of the breast muscles was removed from the fascia and fat and crushed in a blender. Each 3 samples were mixed to create a mixed sample and stored at −20 °C for nutritional analysis (including conventional nutritional compositions, inosine acid, amino acids, and fatty acids) within a month.

### 2.2. Measurement Items and Methods

#### 2.2.1. Basic Meat Quality Parameters

The same part of the right breast muscle was taken to cut off the fascia and fat, and the flesh color, pH, water loss rate and shearing force were measured within 24 h of sampling. The flesh color was measured using a JZ-350 portable color meter (Shenzhen Jinzhun Instrument Equipment Co., Ltd., Shenzhen, China); and the brightness (L*), redness (a*), and yellowness (b*) values were measured in three locations on the surface of the pectoralis major at room temperature; and finally the average value was taken. Twenty-four hours after slaughter, the muscles were minced, and the pH was measured with a Sartorius PB-10 pH meter (German Sartorius, Gottingen, Germany). Before pH measurement, the pH meter was calibrated using calibration buffers (pH 7.0 and pH 5.5) and later adjusted to the meat temperature of 4 °C. The water loss rate was measured using a steel ring allowable dilatometer (Beijing Dadi Keyu Machinery Equipment Co., Ltd., Beijing, China). About 5 g (W1) of fresh muscle was taken, and then placed between 18 layers of water absorption papers on the top and bottom. The steel ring compression apparatus was used to press at 35 kg, and the sample was weighed (W2) after 5 min. The water loss rate = (W1 − W2)/W1 × 100%. The muscle shearing force was measured with a C-LM 3 digital display muscle tenderness meter (developed by Engineering College of Northeast Agricultural University, Harbin, China). Raw meat samples were cut to about 2 cm in length, 1 cm in width, and 1 cm in thickness with a surgical blade in parallel to the direction of the muscle fibers. Each sample was measured 3 times, and the average value was taken.

#### 2.2.2. Conventional Nutritional Compositions

Ten mixed wet samples of each breed were measured for moisture, crude protein, and crude fat. Three replicates were measured for each sample, and finally the average value was taken. A small amount of sea sand was placed in the cleaned dish and dried in a drying box at 103 °C for 30 min ± 1 min. The dish was taken out and placed in a desiccator, cooled to room temperature, and weighed (W0). A 2~5 g sample was weighed in the dish, stirred well, and weighed (W1). The dish was placed in a drying box at 103 °C, dried for 4 h ± 0.1 h, taken out, and weighed (W2). Then, the dish was put in the drying box for 30 min ± 1 min, cooled, and weighed (W3). We made sure that W2 − W3/W1 − W0 < 0.1%, and the moisture content was calculated. The analysis of protein was carried out using the Kjeldahl method (AOAC, 1995) in which the content of total nitrogen was multiplied by a 6.25 coefficient [17]. The fat contents were analyzed and quantified according to the Soxhlet procedure with the Soxtec System HT4 1045 (Tecator, Sweden) (AOAC, 1995) [17].

#### 2.2.3. Inosine Acid

The breast muscle sample was minced with a meat grinder, weighed to 5 g (accurate to 0.0001× *g*), placed in a 50 mL plastic centrifuge tube, added to 20 mL of 6% perchloric acid in portions, and homogenized with a high-speed tissue homogenizer. The homogenate was centrifuged at 8000× *g* r/min for 13 min and filtered in a 100 mL Erlenmeyer flask. The precipitate was homogenized again with 15 mL of 6% perchloric acid and centrifuged, and the supernatants were combined twice. The pH was adjusted to 6.5 with 5.0 mol/L and 0.5 mol/mol/L of NaOH. The fluid under test was filtered with a 0.45 µm filter before determination for analysis with a Water 2695 Alliance high-performance liquid chromatograph (Shimadzu Corporation, Kyoto, Japan). The chromatographic column was a Water Atlantis DG8 (5 μm × 4.6 mm × 150 mm).

#### 2.2.4. Amino Acid

Dry samples (0.1 g) were accurately weighed and hydrolyzed in 15 mL of hydrochloric acid (6 mol/L HCl) in thick-walled test tubes. The tubes were filled with nitrogen, sealed, and then placed in a thermostatic drier oven. The samples were hydrolyzed at (110 ± 1) ℃ for 24 h. The hydrolyzates were transferred and diluted in 50 mL volumetric flasks using 0.02 mol/L of HCl. After mixing, 1 mL of solution was transferred to a 6 cm glass dish and evaporated to dryness in a water bath at 65 °C. An L-8900 amino acid automatic analyzer (Hitachi High-tech Co., Ltd., Shanghai, China) was used to determine the amino acid content of the samples and record the data. After dissolving in 2 mL of HCl (0.02 mol/L) and filtering (0.22 μm), amino acids were fractionated on a protein hydrolysate analysis column (HISCO-855-4506, Hitachi) at an outflow rate of 0.40 mL/min. The individual amino acids were identified via comparison with standards.

#### 2.2.5. Fatty Acid

Ground samples (2 g) were mixed with a chloroform and methanol (2:1; *v*/*v*) solution. The ampoules were sealed in the flame of a gas burner and placed in a thermostat (105 °C) for 2 h. The obtained supernatant was used to prepare the fatty acid methyl esters (FAME) by using a methanol/sulfuric acid mixture (95:5) and hexane following the esterification process outlined earlier. The measurement was performed using a 2010Plus gas chromatograph (Shimadzu Corporation, Kyoto, Japan). The chromatographic column was an SPTM-2560 (0.20 μm × 0.25 mm × 100 m). The column temperature was held at 50 °C for 1 min, and then the temperature was raised to 150 °C at a rate of 15 °C per min. The temperature was later increased to 175 °C at a rate of 2.50 °C and held for 5 min and finally increased to 220 °C at a rate of 2.50 °C per min and kept for 5 min. The injector and detector temperatures were 225 and 250 °C, respectively. The carrier gas was helium (flow rate: 1.2 mL/min). Fatty acids were identified by comparing the retention times of methyl easters in the analyzed samples and the standard mixture of fatty acid methyl esters (Sigma, St. Louis, MO, USA).

### 2.3. Statistical Analysis

Data were collated in Excel 2003, and SPSS 16.0 software was used to perform single-factor analysis of variance to test the significance of differences between the groups; *p* < 0.05 was considered significant. Data are expressed as mean ± standard deviation.

## 3. Results

### 3.1. Comparison of Basic Meat Quality Parameters in Breast Muscle among Different Breeds of Pigeons

The comparison of the basic meat quality parameters in breast muscle among different breeds of pigeons is shown in Table 1. The results showed that there were significant differences in flesh color (L*, b*), pH, and water loss rate of different breeds of pigeons (*p* < 0.05). Compared with White King pigeons, the local breeds (Taihu pigeon, Shiqi pigeon, Tarim pigeon, and Boot pigeon) had dark breast meat and a low water loss rate, while White King pigeon meat was yellowish.

### 3.2. Comparison of Conventional Nutritional Compositions between Different Breeds of Pigeons

The comparison of the conventional nutritional compositions among different breeds of pigeons is shown in Table 2. The results showed that there was no significant difference in water and fat content in the breast muscles of different breeds of pigeons (*p* > 0.05). The protein content of the breast muscles of Taihu, Tarim, and Shiqi pigeons was significantly higher than that of White King pigeons (*p* < 0.05). The Taihu pigeon had the highest protein content, reaching 22.72%.

### 3.3. Comparison of Inosinic Acid Content between Different Breeds of Pigeons

**Figure 1:**
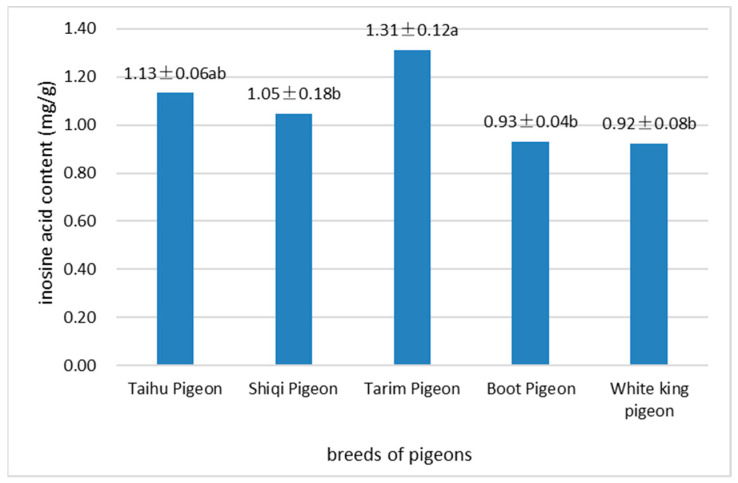
Comparison of the inosine acid content between different breeds of pigeons. Note: Different letters indicate significant differences (*p* < 0.05).

The comparison of the inosinic acid content between different breeds of pigeons is shown in Figure 1. The results showed that the inosinic acid content of the breast muscles of local breed pigeons was higher than that of White King pigeons; among them, Tarim pigeons had the highest inosinic acid content (1.31 mg/g), which was significantly higher than that of Shiqi pigeons, Boot pigeons and White King pigeons (*p* < 0.05). The inosinic acid content of Taihu pigeons (1.13 mg/g) was higher than that of Shiqi, Boot, and White King pigeons but lower than that of Tarim pigeons.

### 3.4. Comparison of Amino Acid Contents among Different Breeds of Pigeons

The comparison of the amino acid contents among different breeds of pigeons is shown in Table 3. The results showed that there was no significant difference in the content of amino acids, the ratio of essential amino acids, and the ratio of umami amino acids in the breast muscles of different breeds of pigeons (*p* > 0.05). Local breeds (Taihu pigeons, Shiqi pigeons, Tarim pigeons, and Boot pigeons) had a slightly higher proportion of essential amino acids than White King pigeons but not at a significant level (*p* > 0.05), while the proportion of umami amino acids was also slightly lower than that of White King pigeons (*p* > 0.05).

### 3.5. Comparison of Fatty Acid Contents between Different Breeds of Pigeons

The comparison of fatty acid content in breast muscles of different breeds of pigeons is shown in Table 4. The results showed that the percentage of saturated fatty acids (SFAs) in the breast muscles of local breeding pigeons was significantly lower than that of White King pigeons (*p* < 0.05), and the percentages of lauric acid, palmitic acid, eicosanoic acid, behenic acid, and behenic acid in SFAs reached significant levels (*p* < 0.05). Taihu pigeons had the highest percentage of monounsaturated fatty acids (MUFAs) (44.58%), which was significantly higher than that of other breeds (*p* < 0.05). The proportion of polyunsaturated fatty acids (PUFAs) in Shiqi pigeons and Tarim pigeons was significantly higher than that in other breeds (*p* < 0.05). The content of eicosapentaenoic acid (EPA) in the breast muscles of Taihu pigeons was significantly higher than that in other breeds (*p* < 0.05).

## 4. Discussion

The conventional physical traits of meat quality, which include flesh color, pH value, hydraulic power, water loss rate, etc., reflect the appearance and internal quality of muscles [18]. Flesh color does not contribute much to muscle flavor, but it has been regarded as a flesh-quality trait mainly because it is affected by the meat’s protein structure, myoglobin chemical state, and lipid oxidation [19]. Compared with White King pigeons, the local breeds (Taihu pigeons, Shiqi pigeons, Tarim pigeon, and Boot pigeon) had darker and less yellow breast meat, which may have been related to the amount of protein, fat, and minerals deposited in muscle [20]. Shear force is an important index to measure the tenderness of poultry; the lower the shear force is, the more tender the meat. The water loss rate is a commonly used index to reflect the nutrition and texture of muscle and the taste and juiciness of meat products during processing [21]. Ye et al. [22] showed that the Warner–Bratzler shear force was not significantly different between Shiqi pigeons and White King pigeons, while the moisture content of Shiqi pigeons was significantly lower than that of White King pigeons. The results of this study were similar to those of Ye’s study, and there were no significant differences in shear force between White King pigeons and the local breeds (Taihu pigeon, Shiqi pigeon, Tarim pigeon, and Boot pigeon); however, compared with White King pigeons, the local breeds had a low water loss rate, which is an advantage.

Conventional chemical components such as moisture, organic matter, crude protein, crude fat, and crude ash in poultry muscle are basic indices used to evaluate the nutritional value of meat [23]. Pigeons, which have a high nutritional value, low fat content, and high digestibility, are superior due to their chemical composition [24]. Pomianowski et al. [15] tested the chemical composition of three pigeon breeds (Wroclawski, King, and Europigeon); the moisture content was 66.52–70.59%, the protein content was 21.73–23.61%, the fat content was 4.32–7.07%, and King pigeons had the lowest fat content. In this study, the protein contents were close to those in the study by Pomianowski, but the fat contents of all pigeon varieties we tested were lower than those in Pomianowski’s study, possibly because the chemical components of pigeon meat are closely related to the nutrient supply of feed. Compared with White King pigeons, the local breeds had higher protein and lower fat contents under the same feeding conditions (Taihu pigeons had the highest protein content), which means that the nutrient compositions of the meat of Chinese local breeds are more optimized.

In recent years, the palatability of meat has received increasing attention. The taste-active components in meat extract include free glutamic acid, 5′-inosinic acid, and potassium ions [25]. Inosinic acid, a hypoxanthine nucleotide, has an important influence on the water-holding capacity, physical properties, and sensory properties of meat; it plays an important role in meat flavor and has been regarded as an important index to measure the freshness of meat worldwide [26]. The main factors affecting the inosinic acid content of meat are varieties, feed additives, dietary nutrient composition, feeding methods, storage conditions, and so on [27,28]. Li et al. [29] believed that inosinic acid was an essential index in the meat quality evaluation system of squabs, and the results of their study showed that the inosinic acid in White King pigeon meat was the highest compared with that of Silver King pigeons, European pigeons, Tyson pigeons, and Suwei pigeons. In this study, the inosinic acid in the meat of local Chinese breeds was still higher than that in the meat of White King pigeons, which means that the meat of local Chinese pigeon breeds has a better flavor and is more popular with consumers.

Amino acids are the basic units of proteins, and the nutritional value of proteins is closely related to the content and structure ratio of amino acids in proteins [30]. The quality of meat proteins is determined by the type and content of amino acids, and EAAs are the most important indices to evaluate the nutritional level of proteins [31]. EAAs play an important role in maintaining health and metabolism. The concentration of EAAs and the EAA/NEAA ratio determines the nutritional value of meat protein [32]. Generally, those amino acids in meat are considered as the precursors of flavoring substances that can react with soluble reducing sugars such as glucose and fructose to form flavoring substances [33]. Amino acid contents, the percentage of essential amino acids, and umami amino acids in the meat of the analyzed pigeons did not differ statistically in our study. This might have been because the effect of feed composition on the amino acid composition of meat was higher than the variety effect. Under the same feeding conditions, the percentage of essential amino acids of Chinese local pigeon breeds was slightly higher than that of White King pigeons, so the type and content of amino acids of Chinese local pigeon meat are more optimized.

Fatty acids are important substances for maintaining cell membrane integrity and chemical transmission and have functions such as promoting central nervous system development, generating energy, transmitting oxygen, and regulating inflammatory mechanisms [34]. However, meat fat containing high proportions of SFAs has been perceived as detrimental to human health and may cause cholesterol levels to increase, indirectly leading to cardiovascular diseases, obesity, and cancer [35]. Huang et al. [32] tested the intramuscular fatty acid composition among three Chinese indigenous pig breeds and found that the percentage of SFAs in those meats was approximately 38.4–40.2%. Valentini et al. [36] tested the meat of broilers fed glycerol monolaurate additives and found that the total SFA content was approximately 31.02–34.70%. Aidyn et al. [37] found that the percentage of SFA in female turkey muscles in Kazakhstan ranged from 50.67% to 52.64%. In this study, the percentage of SFAs in pigeon meat was similar to that in the meat of broilers [36], and the percentage of SFAs in Chinese local pigeon meat was significantly lower than that in White King pigeon meat, which suggested that Chinese local pigeon meat is better for human health. In addition, the content of EPA in the meat of Taihu pigeons was significantly higher than that in the meat of other breeds and 62.07% higher than that in the meat of the lowest booted pigeon, which is beneficial for young children’s brain development and prevention of cardiovascular and cerebrovascular diseases.

## 5. Conclusions

Compared with White King pigeons, the breast meat of local breed pigeons (Taihu pigeons, Shiqi pigeons, Tarim pigeons and Boot pigeons) had dark flesh, good water retention, high protein and inosine contents, a high proportion of essential amino acids, and a low saturated fatty acid ratio. In addition, Taihu pigeons had the highest levels of protein (22.72%), monounsaturated fatty acid (44.58%), and EPA (0.47%) contents compared to other breeds. According to this study, the characteristics of different breeds of pigeon meat can be further explored. At the same time, local pigeon varieties can be used as materials to cultivate matching lines with excellent meat quality.

## Figures and Tables

**Table 1:** Comparison of basic meat quality parameters between different breeds of pigeons.

Breeds		Taihu	Shiqi	Tarim	Boot	White King
Meat color	L*	37.40 ± 1.28 ^ab^	36.48 ± 2.07 ^b^	36.31 ± 1.83 ^b^	36.03 ± 1.63 ^b^	38.54 ± 1.91 ^a^
	a*	11.31 ± 0.95	11.41 ± 1.25	10.62 ± 1.22	10.81 ± 1.11	11.30 ± 0.65
	b*	3.99 ± 0.96 ^b^	4.04 ± 0.92 ^b^	3.28 ± 1.57 ^b^	4.12 ± 1.38 ^ab^	5.14 ± 0.62 ^a^
pH value		5.20 ± 0.09 ^c^	5.31 ± 0.10 ^bc^	5.21 ± 0.13 ^c^	5.53 ± 0.18 ^a^	5.37 ± 0.21 ^b^
Shear force (kg)		1.10 ± 0.25	1.21 ± 0.31	1.16 ± 0.26	0.97 ± 0.14	1.03 ± 0.17
Water loss rate (%)		25.58 ± 3.62 ^b^	22.55 ± 2.26 ^c^	24.18 ± 3.36 ^bc^	26.51 ± 2.81 ^ab^	29.20 ± 2.81 ^a^

Note: L*-100 is white, L*-0 is dark (the larger the L* value, the whiter the color); a* > 0 means red degree, a* < 0 means green degree; b* > 0 means yellow degree, b* < 0 indicates the degree of blue. Different letters indicate significant differences (*p* < 0.05).

**Table 2:** Comparison of conventional nutritional compositions between different breeds of pigeons.

Breeds	Taihu	Shiqi	Tarim	Boot	White King
Moisture (%)	75.55 ± 0.40	73.68 ± 1.65	76.48 ± 3.79	75.62 ± 0.51	76.86 ± 1.74
Crude protein (CP) (%)	22.72 ± 0.94 ^a^	21.79 ± 0.19 ^a^	22.69 ± 1.07 ^a^	21.62 ± 0.02 ^ab^	20.01 ± 0.67 ^b^
Crude fat (EE) (%)	2.20 ± 0.24	2.52 ± 0.62	2.15 ± 0.12	2.31 ± 0.32	2.89 ± 0.06

Note: Different letters indicate significant differences (*p* < 0.05).

**Table 3:** Comparison of amino acid contents between different breeds of pigeons (%).

Breeds		Taihu	Shiqi	Tarim	Boot	White King
Essential amino acids	Isoleucine (Lle)	1.65 ± 0.02	1.67 ± 0.03	1.68 ± 0.03	1.64 ± 0.03	1.62 ± 0.04
	Leucine (Leu)	3.00 ± 0.06	3.04 ± 0.06	3.06 ± 0.08	2.98 ± 0.06	2.97 ± 0.07
	Lysine (Lys)	20.07 ± 0.35	20.29 ± 0.30	20.40 ± 0.34	19.99 ± 0.34	19.63 ± 0.48
	Methionine (Met)	1.80 ± 0.02	1.79 ± 0.03	1.81 ± 0.03	1.77 ± 0.03	1.75 ± 0.04
	Phenylalanine (Phe)	1.84 ± 0.03	1.87 ± 0.03	1.89 ± 0.04	1.84 ± 0.03	1.86 ± 0.03
	Threonine (Thr)	4.93 ± 0.08	5.03 ± 0.04	4.65 ± 0.49	4.95 ± 0.14	4.90 ± 0.14
	Tryptophan (Trp)	0.00 ± 0.00	0.00 ± 0.00	0.00 ± 0.00	0.00 ± 0.00	0.00 ± 0.00
	Valine (Val)	1.90 ± 0.04	1.93 ± 0.04	1.94 ± 0.06	1.88 ± 0.03	1.88 ± 0.06
Non-essential amino acids	Histidine (His)	0.94 ± 0.01	0.96 ± 0.02	0.97 ± 0.01	0.95 ± 0.01	0.99 ± 0.01
	Arginine (Arg)	33.24 ± 1.14	32.40 ± 0.98	32.61 ± 1.03	33.05 ± 1.02	33.38 ± 1.27
	Alanine (Ala)	6.17 ± 0.13	6.30 ± 0.11	6.33 ± 0.23	6.22 ± 0.09	6.24 ± 0.11
	Asparaginic acid (Asp)	3.34 ± 0.08	3.40 ± 0.02	3.19 ± 0.28	3.34 ± 0.07	3.33 ± 0.11
	Glutamic acid (Glu)	5.56 ± 0.06	5.61 ± 0.06	5.49 ± 0.08	5.57 ± 0.09	5.53 ± 0.12
	Glycine (Gly)	6.87 ± 0.33	6.99 ± 0.30	7.22 ± 0.52	7.13 ± 0.14	7.11 ± 0.33
	Cystine (Cys)	0.88 ± 0.05	0.82 ± 0.05	0.90 ± 0.13	0.84 ± 0.06	0.92 ± 0.03
	Proline (Pro)	5.10 ± 0.03	5.14 ± 0.08	5.17 ± 0.10	5.10 ± 0.06	5.12 ± 0.10
	Serine (Ser)	1.45 ± 0.02	1.47 ± 0.01	1.38 ± 0.12	1.46 ± 0.05	1.47 ± 0.04
	Tyrosine (Tyr)	1.30 ± 0.02	1.31 ± 0.02	1.31 ± 0.03	1.29 ± 0.02	1.29 ± 0.02
Percentage of essential amino acids		35.18 ± 0.55	35.61 ± 0.46	35.43 ± 0.38	35.04 ± 0.61	34.62 ± 0.84
Percentage of umami amino acids		55.16 ± 0.66	54.70 ± 0.57	54.84 ± 0.38	55.31 ± 0.79	55.59 ± 0.96

Note: Essential amino acids include isoleucine, leucine, lysine, methionine, phenylalanine, threonine, tryptophan, and valine. The umami amino acids include aspartic acid, glutamic acid, glycine, arginine, and alanine.

**Table 4:** Comparison of fatty acid contents between different breeds of pigeons (%).

Breeds	Taihu	Shiqi	Tarim	Boot Head	White King
Lauric acid (C12:0)	0.22 ± 0.09 ^b^	0.11 ± 0.04 ^b^	0.19 ± 0.13 ^b^	0.17 ± 0.03 ^b^	0.42 ± 0.32 ^a^
Myristic acid (C14:0)	0.32 ± 0.03	0.32 ± 0.03	0.33 ± 0.04	0.36 ± 0.02	0.34 ± 0.06
Tetradecanoic acid (C14:1)	0.10 ± 0.01 ^ab^	0.11 ± 0.02 ^a^	0.10 ± 0.02 ^a^	0.11 ± 0.02 ^a^	0.08 ± 0.02 ^b^
Pentadecanoic acid (C15:1)	0.44 ± 0.10 ^b^	0.38 ± 0.07 ^b^	0.57 ± 0.23 ^b^	0.51 ± 0.13 ^b^	1.23 ± 0.78 ^a^
Palmitic acid (C16:0)	19.56 ± 0.41 ^c^	19.76 ± 0.85 ^bc^	20.42 ± 0.46 ^ab^	19.64 ± 0.91 ^bc^	20.65 ± 0.74 ^a^
Palmitoleic acid (C16:1)	7.13 ± 0.34 ^b^	7.43 ± 0.42 ^b^	7.41 ± 0.97 ^b^	8.23 ± 0.09 ^a^	6.93 ± 0.43 ^b^
Heptadecanoic acid (C17:0)	1.27 ± 0.43	1.11 ± 0.61	0.87 ± 0.68	1.26 ± 0.25	1.44 ± 0.28
Stearic acid (C18:0)	9.14 ± 0.64	8.99 ± 0.82	9.55 ± 0.44	9.50 ± 0.34	9.88 ± 0.66
Oleic acid (C18:1)	32.46 ± 3.40 ^a^	29.01 ± 1.42 ^b^	27.17 ± 0.22 ^b^	29.27 ± 1.34 ^b^	27.66 ± 0.60 ^b^
Linoleic acid (C18:2)	23.32 ± 3.25 ^c^	26.92 ± 0.68 ^a^	26.53 ± 1.24 ^ab^	24.50 ± 1.02 ^bc^	24.13 ± 0.98 ^c^
Linolenic acid (C18:3)	0.41 ± 0.05	0.33 ± 0.05	0.37 ± 0.06	0.43 ± 0.09	0.41 ± 0.04
Eicosanoic acid (C20:0)	0.19 ± 0.08 ^b^	0.23 ± 0.07 ^b^	0.25 ± 0.06 ^b^	0.21 ± 0.03 ^b^	0.66 ± 0.54 ^a^
Eicosenoic acid (C20:1)	0.26 ± 0.05 ^b^	0.23 ± 0.02 ^b^	0.23 ± 0.06 ^b^	0.22 ± 0.04 ^b^	0.34 ± 0.04 ^a^
Heneicosanoic acid C21:0	0.28 ± 0.08 ^b^	0.28 ± 0.06 ^b^	0.28 ± 0.12 ^b^	0.31 ± 0.07 ^b^	0.51 ± 0.08 ^a^
Behenic acid (C22:0)	0.20 ± 0.05 ^b^	0.18 ± 0.04 ^b^	0.19 ± 0.06 ^b^	0.22 ± 0.04 ^b^	0.44 ± 0.30 ^a^
Docosaenoic acid (C22:1)	4.01 ± 0.45 ^b^	3.99 ± 0.65 ^b^	4.76 ± 0.08 ^a^	4.42 ± 0.25 ^ab^	4.13 ± 0.42 ^b^
Eicosapentaenoic acid (C20:5)	0.47 ± 0.07 ^a^	0.30 ± 0.05 ^c^	0.33 ± 0.03 ^c^	0.29 ± 0.03 ^c^	0.38 ± 0.03 ^b^
Tecosenoic acid (C24:1)	0.20 ± 0.02 ^c^	0.24 ± 0.03 ^bc^	0.38 ± 0.05 ^a^	0.29 ± 0.09 ^b^	0.25 ± 0.07 ^bc^
Docosahexaenoic acid (C22:6)	0.04 ± 0.04	0.09 ± 0.02	0.09 ± 0.10	0.09 ± 0.01	0.12 ± 0.03
Saturated fatty acids (SFAs)	31.18 ± 0.77 ^bc^	30.98 ± 1.11 ^c^	32.06 ± 0.13 ^b^	31.66 ± 0.64 ^bc^	34.33 ± 1.05 ^a^
Monounsaturated fatty acids (MUFAs)	44.58 ± 3.36 ^a^	41.39 ± 0.98 ^bc^	40.63 ± 1.25 ^c^	43.04 ± 1.53 ^ab^	40.62 ± 1.52 ^c^
Polyunsaturated fatty acids (PUFAs)	24.24 ± 3.18 ^b^	27.63 ± 0.76 ^a^	27.32 ± 1.2 ^a^	25.30 ± 0.92 ^b^	25.05 ± 1.02 ^b^

Note: Different letters indicate significant differences (*p* < 0.05).

## Data Availability

The data presented in this study are available on request from the corresponding author. The data are not publicly available due to commercial restrictions.
